# Prognostic value of HSP27 in 28-day mortality in septic ICU patients: a retrospective cohort study

**DOI:** 10.3389/fmed.2024.1513788

**Published:** 2025-01-09

**Authors:** Lihua Yao, Zaiwei Fan, Fangyi Yao, Xiaozhong Wang

**Affiliations:** ^1^Jiangxi Province Key Laboratory of Immunology and Inflammation, Jiangxi Provincial Clinical Research Center for Laboratory Medicine, Department of Clinical Laboratory, The Second Affiliated Hospital, Jiangxi Medical College, Nanchang University, Nanchang, Jiangxi, China; ^2^Department of Clinical Laboratory, Affiliated Hospital of North Sichuan Medical College, Nanchong, Sichuan, China; ^3^Department of Orthopedics, The Second Affiliated Hospital, Jiangxi Medical College, Nanchang University, Nanchang, Jiangxi, China

**Keywords:** HSP27, sepsis, prognosis, infection, inflammatory

## Abstract

**Background:**

This study aimed to investigate the association between serum heat shock protein 27 (HSP27) levels and 28-day mortality in patients with sepsis.

**Methods:**

This retrospective study analyzed the clinical data of 76 septic patients admitted to the intensive care unit (ICU). Fifty non-septic ICU patients and 50 healthy individuals served as control groups. Serum HSP27 levels were measured on the day of ICU admission and compared to sepsis severity and survival outcomes.

**Results:**

Median serum HSP27 levels in septic patients (4.70 ng/mL, IQR: 2.10–13.48 ng/mL) were significantly higher than those in both non-septic ICU controls and healthy controls (all *p* < 0.05). Moreover, non-survivors exhibited significantly higher median HSP27 levels (9.30 ng/mL, IQR: 3.62–25.91 ng/mL) compared to survivors (3.03 ng/mL, IQR: 1.48–7.39 ng/mL, *p* < 0.05). Multivariate logistic regression analysis confirmed the association between HSP27 levels and 28-day mortality in sepsis patients. Receiver operating characteristic (ROC) curve analysis revealed an area under the curve (AUC) of 0.720 (95% CI: 0.605–0.817, *p* < 0.001) for HSP27 in predicting sepsis prognosis. Survival analysis demonstrated that patients with high serum HSP27 levels (≥2.61 ng/mL) had a worse prognosis than those with low levels (<2.61 ng/mL).

**Conclusion:**

HSP27 shows potential as a biomarker for the diagnosis and prognosis of sepsis, however, further research is necessary to solidify its clinical utility.

## 1 Introduction

As a systemic inflammatory response syndrome triggered by infection, the definition and diagnostic criteria of sepsis have undergone several revisions in recent years (1). The latest definition, proposed by Sepsis-3 in 2016, describes sepsis as a “life-threatening organ dysfunction caused by a dysregulated host response to infection” (2). A global cohort study estimated that approximately 49 million cases of sepsis occurred worldwide in 2017, resulting in 11 million deaths related to sepsis, which accounted for 19.7% of all global deaths (3). The high incidence and mortality rate of sepsis pose a significant burden on society, necessitating further measures to improve sepsis prevention and treatment. Existing diagnostic indicators have significant limitations in the early detection of sepsis (4). Traditional biomarkers such as C-reactive protein (CRP) and procalcitonin (PCT) are widely used, but their limited sensitivity and specificity hinder their clinical utility (5). Consequently, there is an urgent need to identify novel biomarkers to improve the accuracy of sepsis diagnosis and facilitate early detection.

HSP27, also known as heat shock protein family B (small) member 1 (HSPB1) (6), is a critical stress protein that protects cells from damage (7). It is now understood that the molecular structure of HSP27 exists in both phosphorylated and polymerized states (8). During cellular stress responses, HSP27 plays a key protective role. When cells are exposed to heat, oxidative stress, or inflammatory factors, HSP27 levels are rapidly upregulated, inhibiting protein aggregation and misfolding, thus protecting cells from damage (9).

As a crucial stress protein, HSP27 has demonstrated significant diagnostic and prognostic utility in various diseases (10–13). While studies have shown correlations between other heat shock proteins, such as HSP70, HSP60, and HSP90α, and sepsis severity (14–18), the relationship between HSP27 and sepsis mortality remains uncertain. Therefore, this study aims to assess the prognostic value of HSP27 as a biomarker for sepsis-related mortality.

## 2 Materials and methods

### 2.1 Ethics statement

Ethical approval for this study was obtained from the Ethics Committee of the Second Affiliated Hospital of Nanchang University. All patients provided written informed consent.

### 2.2 Study design

This retrospective case–control study enrolled septic patients admitted to the Second Affiliated Hospital of Nanchang University between September 2022 and September 2023 who met the Sepsis 3.0 diagnostic criteria (19). Patients were categorized into survival and death groups based on their 28-day survival outcomes. Exclusion criteria included HIV infection, immune system disorders, immunosuppressive or cytotoxic drug use, pregnancy, breastfeeding, refusal of informed consent, age under 18 or over 80 years, acute cerebrovascular or cardiovascular incidents, malignant tumors, severe hematological disorders, and ambiguous medical histories affecting SOFA score assessment. Fifty healthy individuals and 50 non-septic intensive care unit (ICU) patients, matched for age and gender with the septic ICU patients, served as the health control and ICU control groups, respectively. The primary reasons for ICU admission among the 76 septic patients were shock, respiratory failure, high-risk surgery, renal failure, acute pancreatitis, and other critical conditions. The 50 non-septic ICU patients were admitted primarily due to respiratory failure (*n* = 27), disturbance of consciousness (*n* = 12), shock (*n* = 7), and other critical conditions (*n* = 4).

### 2.3 Data collection

A retrospective case–control study was conducted using the Hospital Information System (HIS) to retrieve clinical data from patients fulfilling predefined inclusion and exclusion criteria. Access to this data was granted for research purposes. While the authors had access to personally identifiable information during or post-data collection, all data were handled confidentially. General patient demographics, such as age, gender, infection source, comorbidities, length of hospital stay, and vital signs were recorded. Laboratory investigations included peripheral venous blood samples drawn within 48 h of admission to measure complete blood count, liver and kidney function tests, CRP, PCT, and SOFA score. Additionally, venous blood specimens were collected within 2 days of admission to assess complete blood count, liver and kidney function tests, CRP, interleukin-1 beta (IL-1β), PCT, and SOFA score.

### 2.4 Measurement of HSP27 levels

From all participants, a 5 mL fasting blood sample was drawn within 48 h of admission to the ICU. The serum was isolated and stored at −80°C until analysis. Serum HSP27 concentrations were quantified using a human HSP-27/HSPB1 ELISA Kit (Sangon Biotech). Standards, controls, and serum samples were diluted with sample buffer and added to a 96-well microplate. A horseradish peroxidase-conjugated antibody was subsequently added and incubated at 37°C for 60 min. Color development was induced with tetramethylbenzidine (TMB), and the optical density (OD) at 450 nm was measured using a microplate reader. The concentration of HSP27 in each sample was determined by interpolation from a standard curve generated from the OD values of the standard samples.

### 2.5 Statistical analysis

Data entry and statistical analysis were conducted utilizing SPSS software version 25.0 (IBM Corp., Armonk, NY, United States). Categorical variables were assessed with the *χ*^2^ or Fisher’s exact test, depending on sample size considerations. Continuous data were evaluated for normality using appropriate tests. Non-normally distributed data were presented as median and interquartile range (IQR), while normally distributed data were presented as mean and standard deviation. Group comparisons for continuous data were performed using either the Mann–Whitney *U* test for two independent groups or one-way analysis of variance (ANOVA) with *post hoc* tests for multiple comparisons, depending on the presence of equal variances among groups. Receiver operating characteristic (ROC) curves were generated to assess the sensitivity and specificity of HSP27 in diagnosing sepsis. The area under the ROC curve (AUC) with its corresponding 95% confidence interval (CI) was used to evaluate the prognostic value of HSP27. Survival curves were constructed using the Kaplan–Meier method to compare survival times between patient groups. Data visualizations were created using the online tool Hiplot Pro.^1^ A *p*-value of less than 0.05 was considered statistically significant.

## 3 Results

### 3.1 Characteristics of the study population

This study enrolled a total of 176 participants, comprising 76 sepsis patients, 50 ICU controls, and 50 healthy controls. Table 1 provides a detailed overview of the demographic and clinical characteristics of the study. Upon admission to the ICU, sepsis patients exhibited significantly elevated heart rate and blood pressure compared to the ICU control group (*p* < 0.05). The median HSP27 level in sepsis patients at admission was 4.70 ng/mL (IQR: 2.10, 13.48), which was significantly higher than that observed in healthy controls (1.06 ng/mL [IQR: 0.22, 2.06]) and non-septic ICU patients (2.6 ng/mL [IQR: 1.12, 6.08]) (all *p* < 0.05). Additionally, compared to the healthy control group, sepsis patients displayed significant alterations in various hematological parameters, including elevated levels of creatinine, blood urea nitrogen, monocytes, and neutrophils, as well as decreased levels of platelets, hematocrit, and lymphocytes (all *p* < 0.05). Compared to the ICU control group, sepsis patients exhibited significantly higher levels of lymphocytes, platelets, and transferrin (all *p* < 0.05). Furthermore, the SOFA score was significantly higher in sepsis patients compared to ICU controls (*p* < 0.05), indicating a greater degree of organ dysfunction in the sepsis group.

**Table 1:** Characteristics of healthy controls, ICU controls, and septic patients.

Parameters	Healthy controls (*n* = 50)	ICU controls (*n* = 50)	Septic patients (*n* = 76)
Patient characteristics			
Age, years	52.5 (46, 61)	57.5 (47, 70.5)	64.5 (52, 71.75)
Male, female	30 (60%), 20 (40%)	34 (68%), 16 (32%)	53 (69.7%), 23 (30.3%)
Underlying disease (*n*, %)			
Hypertension	/	11 (22.0%)	28 (36.8%)^b^
Diabetes	/	3 (6.0%)	14 (87.5%)^b^
COPD	/	7 (14.0%)	24 (31.6%)^b^
Constants			
Temperature	/	36.49 (36.42, 36.56)	36.52 (36.20, 36.85)
Breath_rate, beats/min	/	19.91 (19.28, 20.48)	19.53 (18.28, 21.17)
Heart_rate, beats/min	/	89 (85, 94)	95 (84, 106)^b^
Systolic blood pressure, mmHg	/	110 (107, 116)	90 (83, 96)^b^
Diastolic blood pressure, mmHg	/	70 (64, 76)	60 (53, 67)^b^
Laboratory values			
White blood cells (×10^9^/L)	5.89 (5.29, 6.66)	11.21 (9.42, 15.67)	10.85 (7.76, 14.45)^a^
Neutrophils (×10^9^/L)	3.11 (2.69, 3.77)	8.89 (8.02, 12.76)	9.31 (6.55, 12.59)^a^
Lymphocytes (×10^9^/L)	2.05 (1.80, 2.50)	0.92 (0.68, 1.52)	0.83 (0.53, 1.18)^a, b^
Monocytes (×10^9^/L)	0.40 (0.33, 0.50)	0.59 (0.40, 0.99)	0.63 (0.33, 0.97)^a^
Hematocrit (%)	44.55 (42.13, 49.25)	36.55 (28.45, 41.83)	34.85 (30.25, 40.43)^a^
Platelets (×10^9^/L)	252 (197.75, 286)	192.5 (148.75, 290)	105 (68, 178.25)^a, b^
Blood urea nitrogen (mmol/L)	4.81 (4.10, 5.69)	5.75 (4.12, 8.45)	7.77 (5.19, 15.01)^a, b^
Creatinine (μmol/L)	69.0 (56.5, 84.25)	60.5 (51, 76)	109.5 (66, 219.5)^a, b^
Total bilirubin (μmol/L)	11.9 (9.68, 15.90)	12.4 (8.1, 17.9)	15.15 (9.63, 28.13)^a^
Ferritin (μg/L)	/	752 (448.75, 1434.5)	797 (427, 2008.25)
Transferrin (g/L)	/	1.44 (0.99, 1.72)	1.14 (0.96, 1.42)^b^
Serum iron (μmol/L)	/	5.55 (3.10, 9.40)	5.80 (4.20, 10.50)
Transferrin saturation (%)	/	27.70 (21.05, 33.98)	27.50 (21.60, 33.40)
TIBC (μmol/L)	/	35.6 (26.45, 41.2)	34.2 (29.2, 41.2)
CRP (mg/L)	/	115.83 (66.61, 165.75)	81.86 (39.24, 147.42)
PCT (ng/mL)	/	1.34 (0.71, 2.37)	1.87 (0.78, 8.16)
HSP27 (ng/mL)	1.06 (0.22, 2.06)	2.6 (1.12, 6.08)	4.70 (2.10, 13.48)^a, b^
SOFA score	/	1 (1, 2)	4 (3, 7)^b^
ICU stay (days)	/	10 (6, 19)	11 (7, 17)

“/” represents that the corresponding data is not obtained; “^a^” represents that the comparison of healthy controls with septic patients, *p* < 0.05; “^b^” represents that the comparison of ICU controls with septic patients, *p* < 0.05.

ICU, Intensive Care Unit; COPD, chronic obstructive pulmonary disease; TIBC, total iron binding capacity; CRP, C-reactive protein; PCT, procalcitonin; HSP27, heat shock protein 27; SOFA, sequential organ failure assessment.

### 3.2 Serum HSP27 levels as a potential diagnostic biomarker for sepsis patients

**Figure 1:**
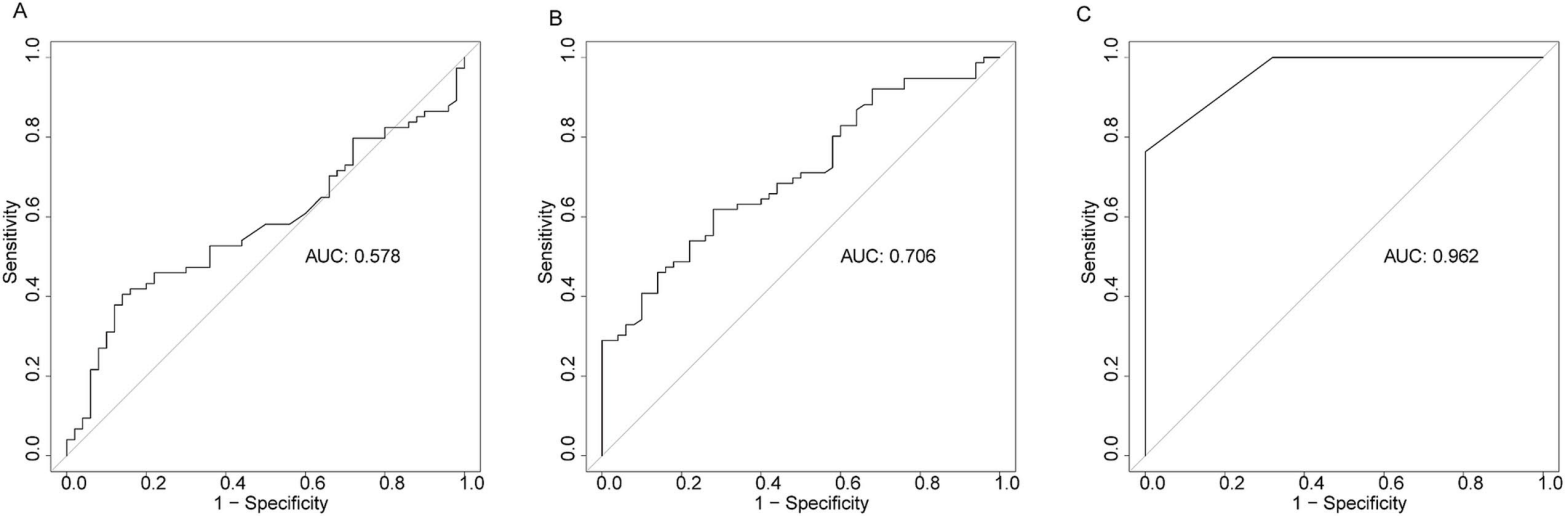
Receiver operating characteristic curve (ROC) of PCT **(A)**, HSP27 **(B)**, and SOFA score **(C)** for diagnosis of sepsis.

To assess the diagnostic performance of HSP27, SOFA score, and PCT in differentiating ICU sepsis patients from ICU controls, we employed ROC curve analysis. HSP27 exhibited an AUC of 0.706 (95% CI: 0.616–0.795, *p* < 0.001), with a sensitivity of 72.0% and a specificity of 61.8%. While this performance was inferior to the SOFA score (AUC: 0.962, 95% CI: 0.940–0.984, sensitivity: 76.3%, specificity: 100%, *p* < 0.001), it surpassed that of PCT (AUC: 0.578, 95% CI: 0.477–0.679, sensitivity: 40.5%, specificity: 86.0%, *p* = 0.088) (Figure 1). These findings suggest that HSP27 may have potential as a diagnostic biomarker for sepsis.

### 3.3 Elevated serum HSP 27 levels are associated with poorer survival outcomes in ICU sepsis patients

Of the 76 sepsis patients, 39.47% (*n* = 30) succumbed to sepsis within 28 days of ICU admission. Table 2 presents a comparative analysis of the baseline characteristics and laboratory parameters between the surviving and non-surviving patient groups. Notably, non-survivors exhibited significantly elevated levels of creatinine, PCT, and IL-1β compared to survivors (all *p* < 0.05). Furthermore, the median HSP27 level was significantly higher in non-survivors [9.30 ng/mL (IQR: 3.62, 25.91)] compared to survivors [3.03 ng/mL (IQR: 1.48, 7.39)] (*p* < 0.001). Besides, non-survivors had significantly higher SOFA scores, indicating a greater degree of organ dysfunction.

**Table 2:** Index comparison between the survival and non-survival groups of septic patients.

Parameters	Survival (*n* = 46)	Non-survival (*n* = 30)	*P-*value
Patient characteristics			
Age, years	64 (49.75, 71)	66 (54.75, 72.25)	0.500
Male/Female	34/12	19/11	0.326
Underlying disease (*n*, %)			
Hypertension	17 (37.0%)	11 (36.7%)	0.980
Diabetes	8 (17.4%)	6 (20.0%)	0.774
COPD	13 (28.3%)	11 (36.7%)	0.441
Laboratory values			
White blood cells (×10^9^/L)	9.79 (5.83, 14.30)	11.53 (10.17, 16.57)	0.079
Neutrophils (×10^9^/L)	8.16 (4.19, 12.58)	10.25 (8.45, 14.04)	0.064
Lymphocytes (×10^9^/L)	0.90 (0.63, 1.26)	0.81 (0.43, 1.14)	0.338
Monocytes (×10^9^/L)	0.58 (0.29, 0.85)	0.78 (0.41, 1.08)	0.118
Hematocrit (%)	34.70 (29.75, 39.90)	35.90 (31.98, 41.48)	0.506
Platelets (×10^9^/L)	105 (77.75, 182)	108 (54, 195.75)	0.780
Blood urea nitrogen (mmol/L)	6.91 (5.13, 11.67)	10.82 (6.09, 19.11)	0.107
Creatinine (μmol/L)	87 (60.25, 174.25)	166.5 (72.75, 279)	0.039
Uric acid (μmol/L)	269 (180, 470)	349 (143.75, 536.5)	0.592
Total bilirubin (μmol/L)	14.85 (9.88, 29.05)	15.65 (9.40, 27.65)	0.927
Ferritin (μg/L)	944 (413.75, 2015.5)	544 (407.75, 1811)	0.476
Transferrin (g/L)	1.14 (0.99, 1.41)	1.15 (0.87, 1.45)	0.596
Serum iron (μmol/L)	5.8 (4.2, 9.75)	5.6 (3.75, 12)	0.864
Transferrin saturation (%)	27.8 (23.5, 33.35)	26.95 (16.9, 35.6)	0.632
TIBC (μmol/L)	34.7 (29.25, 42.15)	32.75 (27.65, 40.15)	0.628
PaO_2_ (mmHg)	75 (52, 98.05)	74.5 (57, 93)	0.863
PaCO_2_ (mmHg)	34 (28.8, 37.5)	35.5 (30.0, 40.1)	0.396
CRP (mg/L)	69.20 (31.26, 126.85)	92.55 (44.00, 203.78)	0.162
PCT (ng/mL)	1.47 (0.75, 4.45)	10.25 (8.45, 10.04)	0.039
HSP27 (ng/mL)	3.03 (1.48, 7.39)	9.30 (3.62, 25.91)	0.001
IL-1β (pg/mL)	2.17 (0.91, 4.60)	4.56 (1.52, 8.05)	0.035
SOFA score	4 (2, 5.25)	7 (3, 10)	<0.001
ICU stay (days)	12 (7, 18)	9 (6, 15.5)	0.183

COPD, chronic obstructive pulmonary disease; TIBC, total iron binding capacity; PaO_2_, pressure of oxygen; PaCO_2_, partial pressure of carbon dioxide; CRP, C-reactive protein; PCT, procalcitonin; HSP27, heat shock protein 27; IL-1β, interleukin 1 beta; SOFA, sequential organ failure assessment; ICU, Intensive Care Unit.

### 3.4 HSP27 levels related to the severity of ICU sepsis patients

**Figure 2:**
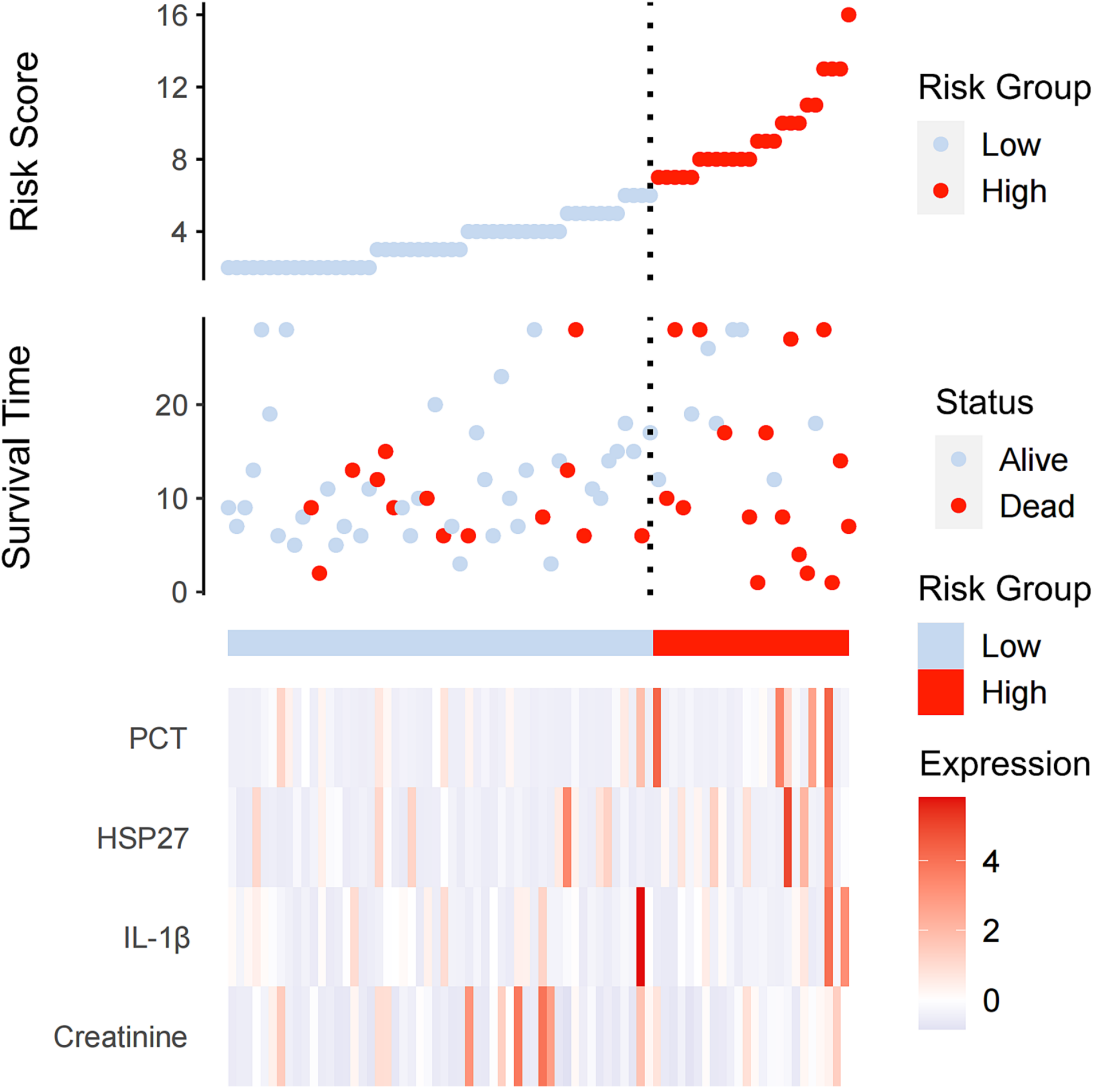
Serum PCT, HSP27, IL-1β, and creatinine levels at admission correlated with disease severity (SOFA score) in septic patients.

To investigate the association between HSP27 levels and sepsis severity, we performed a risk factor analysis. The results indicated that higher HSP27 levels were associated with elevated levels of PCT and SOFA scores (Figure 2). Univariate and multivariate logistic regression models were employed to identify independent predictors of 28-day mortality among septic patients. Univariate analysis revealed a significant positive correlation between higher HSP27 levels and increased 28-day mortality (odds ratio [OR] = 1.035, 95% confidence interval [CI]: 1.005–1.067, *p* = 0.022). Similarly, PCT and SOFA scores were significantly associated with increased mortality (OR = 1.160, 95% CI: 1.046–1.265, *p* = 0.001; and OR = 1.348, 95% CI: 1.130–1.608, *p* = 0.001, respectively). In contrast, creatinine levels were not significantly associated with 28-day mortality (OR = 1.002, 95% CI: 0.999–1.005, *p* = 0.122).

To further elucidate the independent prognostic value of HSP27, multiple logistic regression models were constructed (Table 3). After adjusting for potential confounders such as creatinine, PCT, and IL-1β, HSP27 remained significantly associated with 28-day mortality in sepsis (Model 2: OR = 1.038, 95% CI: 1.007–1.071, *p* = 0.017; Model 3: OR = 1.039, 95% CI: 1.006–1.073, *p* = 0.021; Model 4: OR = 1.036, 95% CI: 1.003–1.071, *p* = 0.035). However, upon further adjustment for the SOFA score (Model 5: OR = 1.034, 95% CI: 0.998–1.072, *p* = 0.064), the association between HSP27 and mortality lost statistical significance. These findings suggest that HSP27 levels are related to the 28-day mortality of ICU sepsis, but the SOFA score remains the established gold standard in clinical practice.

**Table 3:** Multivariate logistic regression analysis for sepsis after adjusting effects of confounders.

Multivariate logistic regression analysis	*B*	S. E.	Wald	*P*-value	Odds ratio	95%CI
Model 1	0.035	0.015	5.239	0.022	1.035	1.005–1.067
Model 2	0.037	0.016	5.657	0.017	1.038	1.007–1.071
Model 3	0.038	0.016	5.364	0.021	1.039	1.006–1.073
Model 4	0.036	0.017	4.463	0.035	1.036	1.003–1.071
Model 5	0.034	0.018	3.435	0.064	1.034	0.998–1.072

Model 1 included only HSP27. In Model 2, a multivariate analysis was conducted incorporating variables HSP27 and Creatinine (CREA). A multivariate analysis was performed incorporating variables HSP27, CREA and procalcitonin (PCT) in Model 3. Model 4 included HSP27, CREA, PCT and IL-1β. Finally, in Model 5, HSP27, CREA, PCT and IL-1β, and the SOFA score were included for multivariate analysis.

### 3.5 The ability of HSP27 to predict 28-day mortality in sepsis patients

**Figure 3:**
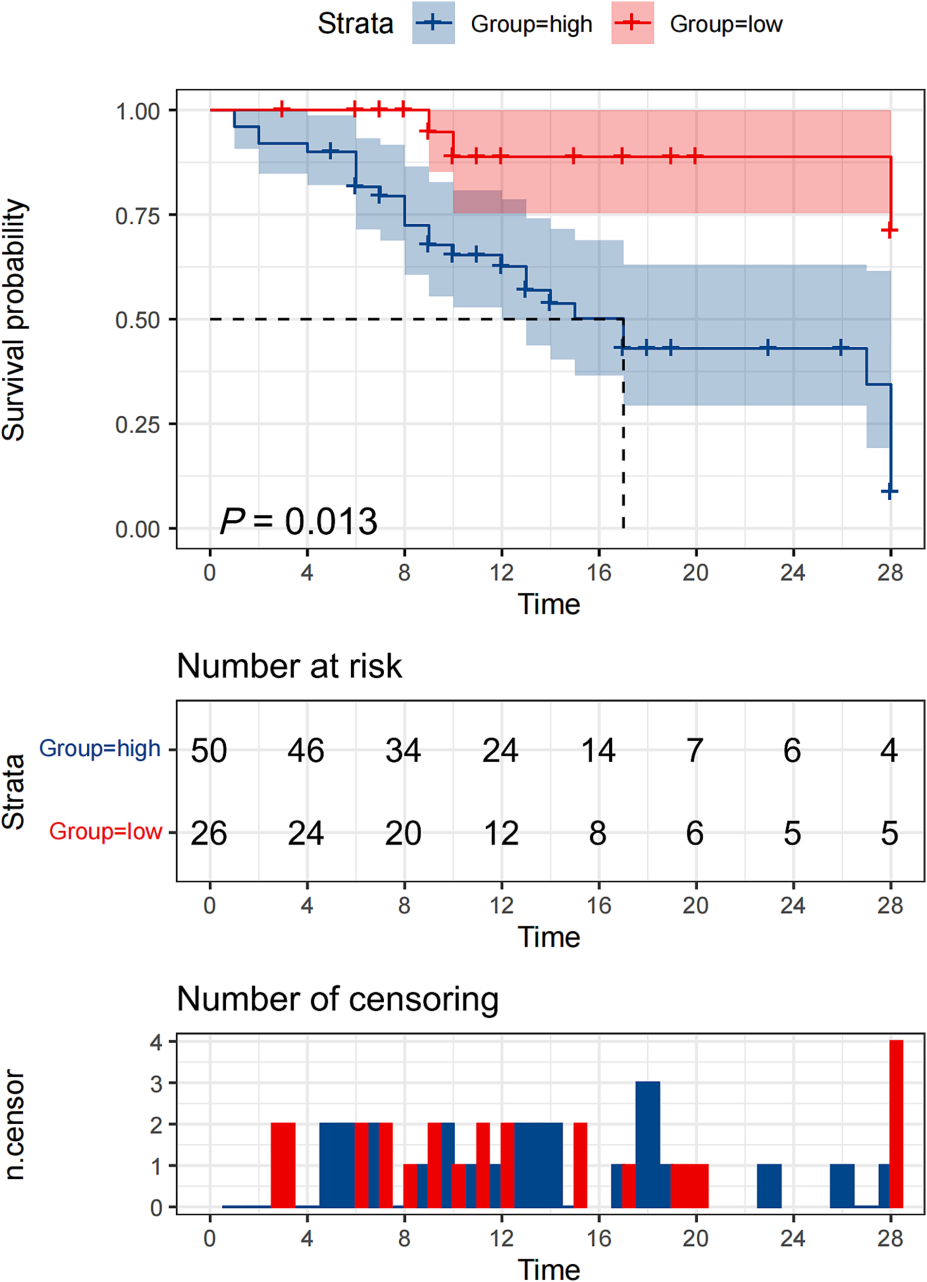
Kaplan–Meier survival curves of 76 adult patients with sepsis based on the HSP27 cutoff value (2.61 ng/mL) on day of ICU admission.

To evaluate the prognostic significance of HSP27 in predicting 28-day survival, ROC curve analysis was performed. The AUC for HSP27 was 0.720 (95% CI: 0.605–0.817, *p* < 0.001), indicating good predictive accuracy. The optimal cutoff value for HSP27 was 2.61 ng/mL, yielding a sensitivity of 90.0% and a specificity of 50.0% in predicting 28-day mortality (Table 4). A combination of HSP27 and the SOFA score further improved prognostic accuracy, with an AUC of 0.767 (95% CI: 0.656–0.856, *p* < 0.001) and a specificity of 91.3%. This combined approach outperformed the use of either SOFA score (AUC: 0.702, 95% CI: 0.607–0.819, specificity: 86.6%) or HSP27 alone. Kaplan–Meier survival curves were constructed to compare the 28-day mortality rates between patients with high and low HSP27 levels based on the established cutoff value. The 28-day survival rate was significantly lower for patients with HSP27 levels above the cutoff than those with lower levels (log-rank test, *p* = 0.013) (Figure 3).

**Table 4:** Receiver operating characteristic (ROC) analysis for laboratory indexes on sepsis prognosis.

Indexes	AUC (95% CI)	Cut-off	*z*-value	Youden index *J*	*P*-value	Sensitivity	Specificity
Creatinine (μmol/L)	0.643 (0.525–0.749)	205	2.136	0.281	0.033	43.3%	84.8%
PCT (ng/mL)	0.613 (0.494–0.722)	3.06	1.619	0.296	0.106	60.0%	69.6%
HSP27 (ng/mL)	0.720 (0.605–0.817)	2.61	3.767	0.400	<0.001	90.0%	50.0%
IL-1β (pg/mL)	0.643 (0.525–0.750)	3.00	2.064	0.317	0.039	60.0%	71.7%
SOFA score	0.722 (0.607–0.819)	6.0	3.631	0.359	<0.001	53.3%	86.6%
HSP27 + SOFA score	0.767 (0.656–0.856)	/	4.748	0.446	<0.001	53.3%	91.3%

AUC, area under curve; CI, confidence interval; PCT, procalcitonin; HSP27, heat shock protein 27; SOFA, sequential organ failure assessment.

## 4 Discussion

As a systemic inflammatory response syndrome, sepsis often presents with non-specific symptoms in its early stages, leading to delayed diagnosis (20). However, the underlying pathological processes of sepsis initiate early, gradually causing systemic damage (21, 22). In its advanced stages, sepsis rapidly progresses, characterized by systemic immune dysregulation, uncontrolled inflammation, and tissue injury. Patients with advanced sepsis may develop multiple organ dysfunction syndrome (MODS), a life-threatening condition that is difficult to manage and associated with poor outcomes (23). Early intervention is critical to implement timely and effective therapeutic strategies before the condition becomes irreversible (24). Therefore, there is an urgent need for a biomarker that can facilitate early diagnosis and prognostication of sepsis.

The SOFA score, a widely recognized standard for assessing organ dysfunction, is a crucial tool in the diagnosis and prognostication of sepsis. Despite its complexity, which involves multiple variables, the SOFA score remains the primary method used in clinical practice to evaluate the severity and predict the outcome of sepsis (25). In recent years, an increasing number of biomarkers, including CRP, PCT, and IL-1β, are being utilized in clinical settings to enhance, have been explored for their potential to improve the predictive accuracy of sepsis prognosis (26–28). However, their limitations in terms of sensitivity and specificity hinder their widespread clinical application in the diagnosis and prognostication of sepsis.

Our study revealed that ICU sepsis patients exhibited significantly elevated serum HSP27 levels within 48 h of ICU admission compared to a control group. As an anti-inflammatory molecule (29), elevated HSP27 plays a crucial protective role in mitigating organ dysfunction induced by sepsis (30–32). To further assess the diagnostic utility of HSP27 in ICU sepsis, ROC curve analysis demonstrated a significantly higher AUC for HSP27 compared to PCT. Consequently, HSP27 emerges as a promising biomarker for ICU sepsis. Moreover, sepsis patients within the mortality group exhibited significantly higher serum HSP27 levels compared to the survival group. Additionally, sepsis patients with elevated serum HSP27 levels at admission exhibited a higher 28-day mortality rate. By combining HSP27 levels with SOFA scores, the specificity of ROC-based diagnosis increased from 86.6 to 91.3% (*p* < 0.001, Table 4). Finally, Kaplan–Meier survival analysis indicated a correlation between elevated serum HSP27 levels and reduced patient survival rates. These findings collectively demonstrate the prognostic value of HSP27 levels in ICU patients with sepsis.

More importantly, while the AUC for HSP27 was slightly lower than that for the SOFA score, the early changes in HSP27 expression levels during the onset of sepsis could facilitate earlier diagnosis and prognostic assessment. Early identification of sepsis patients is crucial for improving patient outcomes, and the early diagnostic potential of HSP27 may contribute to more timely clinical interventions. Moreover, the level of HSP27 correlates with sepsis severity, with higher levels associated with more severe disease and poorer prognosis. This suggests that HSP27 may serve as a valuable prognostic biomarker. Additionally, the quantification of serum HSP27 levels using ELISA technology is a convenient and highly accurate method, making it suitable for widespread implementation in hospital laboratories. By closely monitoring changes in HSP27 levels, clinicians can assess the therapeutic response of sepsis patients and make timely adjustments to their treatment plans.

In conclusion, our findings suggest that HSP27 may serve as a valuable biomarker for both the diagnosis and prognosis of sepsis.

### 4.1 Limitations

Although this retrospective, single-center study offers valuable insights into the role of HSP27 in sepsis, several limitations inherent to its design and scope must be acknowledged. Firstly, the retrospective nature of the study and its single-center design may introduce selection bias, potentially overestimating the diagnostic and prognostic utility of HSP27. To address this, future large-scale, multicenter, prospective studies are imperative. Secondly, the relatively small sample size and the inclusion of predominantly critically ill patients limit the generalizability of the findings to other patient populations, such as those with postoperative or non-critical sepsis. Expanding the sample size and incorporating a broader spectrum of patient conditions would enhance the reliability and applicability of the results. Thirdly, despite efforts to account for known confounders, the possibility of unmeasured or unrecognized confounding factors that may influence the observed associations cannot be entirely excluded. Finally, the lack of serial monitoring of serum HSP27 levels at multiple time points during the course of sepsis precludes a comprehensive understanding of the temporal relationship between HSP27 fluctuations and disease progression. Addressing these limitations through future studies will provide a more comprehensive understanding of the potential clinical utility of HSP27 in sepsis management.

## 5 Conclusion

In summary, this study demonstrates that serum HSP27 levels are significantly elevated in patients with sepsis, particularly in those who do not survive the 28-day period. These findings suggest a strong association between elevated HSP27 levels and increased mortality risk in sepsis patients. Our results highlight the potential utility of HSP27 as a valuable biomarker for the diagnosis, prognosis, and potentially the therapeutic management of sepsis.

## Data Availability

The raw data supporting the conclusions of this article will be made available by the authors, without undue reservation.

## Footnotes

^1^ https://hiplot.com.cn/
